# Effects of Nonthermal Radiofrequency Stimulation on Neuronal Activity and Neural Circuit in Mice

**DOI:** 10.1002/advs.202205988

**Published:** 2023-02-08

**Authors:** Yanhui Hao, Weiqi Liu, Yujie Liu, Ying Liu, Zhengtao Xu, Yumeng Ye, Hongmei Zhou, Hua Deng, Hongyan Zuo, Hong Yang, Yang Li

**Affiliations:** ^1^ Department of Experimental Pathology Beijing Institute of Radiation Medicine Beijing 100850 China; ^2^ Life Science Department Foshan University Foshan 528231 China; ^3^ Academy of Life Science Anhui Medical University Hefei 230032 China; ^4^ Department of Pathology Chengde Medical College Chengde 067000 China

**Keywords:** calcium, memory, mouse, neural circuit, radiofrequency

## Abstract

Whether the nonthermal effects of radiofrequency radiation (RFR) exist and how nonthermal RFR acts on the nervous system are unknown. An animal model of spatial memory impairment is established by exposing mice to 2856‐MHz RFR in the range of thermal noise (≤1 °C). Glutamate release in the dorsal hippocampus (dHPC) CA1 region is not significantly changed after radiofrequency exposure, whereas dopamine release is reduced. Importantly, RFR enhances glutamatergic CA1 pyramidal neuron calcium activity by nonthermal mechanisms, which recover to the basal level with RFR termination. Furthermore, suppressed dHPC dopamine release induced by radiofrequency exposure is due to decreased density of dopaminergic projections from the locus coeruleus to dHPC, and artificial activation of dopamine axon terminals or D1 receptors in dHPC CA1 improve memory damage in mice exposed to RFR. These findings indicate that nonthermal radiofrequency stimulation modulates ongoing neuronal activity and affects nervous system function at the neural circuit level.

## Graphical abstract

The study reports the nonthermal effects and potential mechanisms of radiofrequency‐electromagnetic fields (RF‐EMFs) on the central nervous system in mice. Radiofrequency stimulation can modulate ongoing neuronal activity in vivo through nonthermal mechanisms. In addition, radiofrequency radiation below the body thermal threshold (≤1 °C) can destruct axonal projections and impair cognitive functions.

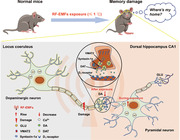

## 1 Introduction

Radiofrequency‐electromagnetic fields (RF‐EMFs, 100 KHz–300 GHz) in the environment have been increasing over the past several decades. Public concern is growing regarding the safety of radiofrequency radiation (RFR), particularly in the microwave frequency range (300 MHz–300 GHz). Many studies have reported the adverse effects of RFR on the brain (and the nervous system in general), including learning and memory impairment,^[^ ^1^ ^]^ sleep disorder,^[^ ^2^ ^]^ anxiety and depression,^[^ ^3^ ^]^ electroencephalography (EEG) changes,^[^ ^4^ ^]^ and even tumors.^[^ ^5^ ^]^ However, studies on the biological effects of RFR are sometimes controversial,^[^ ^6^ ^]^ largely because the neurobiological mechanisms underlying RFR‐induced cognitive impairment remain poorly characterized.

Generally, the toxic effects of RF‐EMFs are classified as thermal or nonthermal effects. After several decades of research, the only substantiated effect of RFR on human health and safety is heating of exposed tissue. Whether nonthermal biological effects exist is debated due to lack of clear and definitive evidence.^[^ ^7^ ^]^ The standards or guidelines for radiofrequency (RF) exposure in the public or occupational sectors, developed by the Institute of Electrical and Electronics Engineers and the International Commission on Non‐Ionizing Radiation Protection, are based on protection against adverse effects that might occur due to increases in tissue or body temperature in excess of 1 °C.^[^ ^7^, ^8^ ^]^ Under these standards, RF‐EMF exposure is usually measured in watts per square meter or watts per kg (determining thermal effect), with modulation models (which might be related to nonthermal effects) not considered. Meanwhile, the adverse effects of nonthermal RFR on the nervous system cannot easily be explained by thermal effects.^[^ ^1^, ^2^, ^3^, ^4^, ^5^ ^]^

Herein, we explored the mechanisms underlying cognitive impairment induced by 2856‐MHz RF‐EMF exposure below the thermal threshold (≤1 °C) at the neuronal activity and neural circuit levels in vivo. Using metal‐free probes, we determined that transcranial RF stimulation may modulate ongoing neuronal activity by nonthermal mechanisms. However, neuronal activity returned to normal with the termination of RF exposure, indicating it does not cause cognitive damage. Furthermore, our study confirmed that long‐distance axonal projections could be important targets sensitive to external RF energy.

## 2 Results

### 2.1 RF Applied in the Study Causes Less Than 1 °C Increase in the Rectal Temperature of Mice

**Figure 1:**
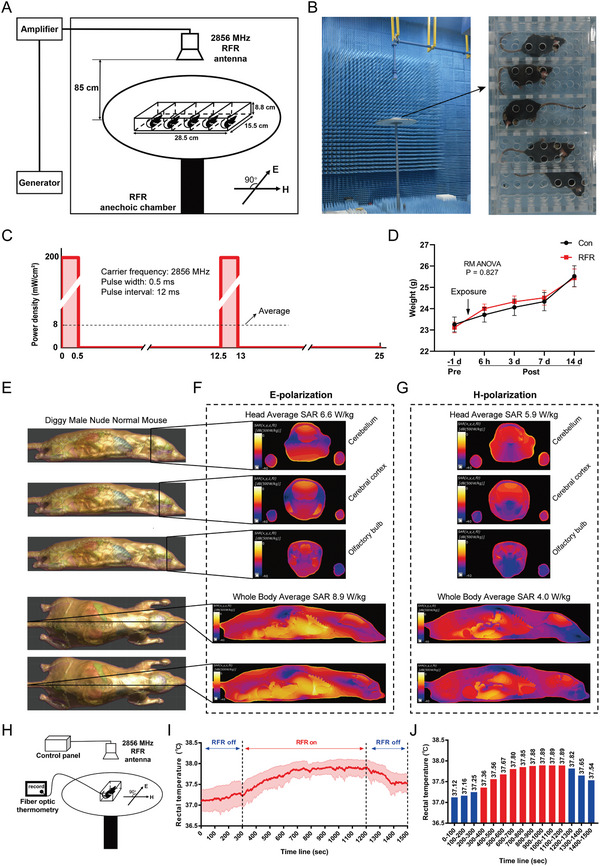
Radiofrequency exposure system and changes in mouse rectal temperature. A,B) Schematic and photos of the radiofrequency (RF) exposure system. E: E‐polarization, H: H‐polarization. C) RF modulation. D) Change in body weight of mice due to RF exposure (*n* = 12 mice). E–G) Specific absorption rate (SAR) maps on Diggy Male Nude Normal Mouse Model simulated by Sim4Life 6.2 software. Diggy Male Nude Normal Mouse (E), SAR maps of E‐polarization (F), SAR maps of H‐polarization (G). H–J) Rectal temperature measured by a fiber optic thermometer (*n* = 3 mice). Schematic of the experimental device (H). Plot of average rectal temperature before, during, and after RF exposure. The red shadow indicates the standard error of the mean (SEM) (I). Histogram of rectal temperature as shown in I (J). Each column represents the rectal temperature average of three biological replicates every 100 s. Data are presented as mean ± SEM. Repeated‐measures analysis of variance was performed to compare the difference between Con and RFR groups (D).

The RF exposure device applied in this study is shown in **Figure** **1**A,B. The mice were fixed in a metal‐free box, arranged along the E‐polarization direction, and placed directly below a 12 dB‐gain horn RF antenna. The RF had a carrier frequency of 2856 MHz, with 80‐Hz square modulation and a duty cycle of 4%. The peak power density at the platform center was 200 mW cm^−2^ and the average power density was 8 mW cm^−2^ (Figure 1C). The power densities were uniform within ±0.4 dB of the mean over the area where the mice were placed. The body weight of the mice was between 23 and 24 g on the day of RF exposure. Pre‐ and post‐radiation weights were measured, and there were no differences between the control (Con) and RFR groups, ensuring that the results were not affected by other physiological conditions (Figure 1D).

Sim4Life (Version 6.2, ZMT, Switzerland) software was used to create simulated anatomical images and specific absorption rate (SAR) maps based on the Diggy Male Nude Normal Mouse Model with a body weight of 23.3 g and length of 8.6 cm (Figure 1E–G). When mice were arranged along the E‐polarization direction of the RF, the main exposure mode in this study, the whole‐body average SAR was calculated to be ≈8.9 W kg^−1^ and the local head SAR was ≈6.6 W kg^−1^ (Figure 1F). If mice faced the H‐polarization direction, the whole‐body average SAR was ≈4.0 W kg^−1^, and the local head SAR was ≈5.9 W kg^−1^ (Figure 1G).

The rectal temperature of the mice was monitored during the RFR treatment using fiber optic thermometry (Figure 1H). The underlying rectal temperature of mice was 37.12 °C, measured under ambient temperature of 17.7 °C and 24% humidity. The rectal temperature of mice increased 0.13 °C before radiation, which may have been caused by the mice struggling. When the RF was turned on, the rectal temperature finally stabilized at 37.89 °C after 600 s of RF exposure, which increased by 0.74 °C in total in relation to the underlying value, as the heat production and dissipation tended to balance (Figure 1I,J). This showed that exposure to 2856 MHz RF with an average power density of 8 mW cm^−2^ applied for 900 s caused less than 1 °C increase in rectal temperature.

### 2.2 RF Exposure Impairs Hippocampus‐Dependent Spatial Learning and Memory in Mice

**Figure 2:**
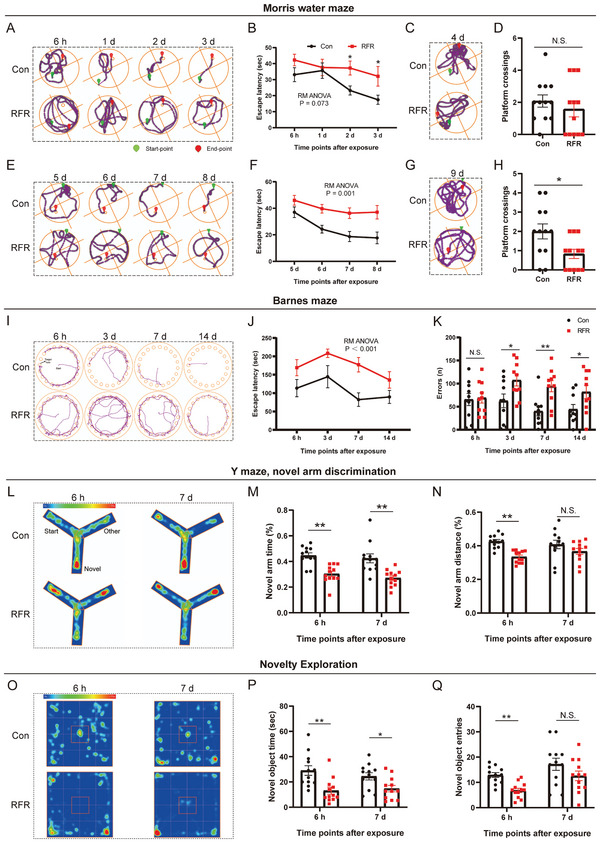
Effects of radiofrequency radiation on cognitive performance in mice. A–H) Morris water maze assays (*n* = 12 mice). Hidden platform tests were conducted 6 h, 1 d, 2 d, and 3 d post exposure, and representative motion trajectory (A) and escape latency (B) are shown. The platform was removed and probe tests were performed, of which representative motion trajectory (C) and platform crossings (D) are shown. E–H) Thereafter, the platform was relocated to the opposite quadrant, and spatial reversal tests were conducted. Representative motion trajectory (E) and escape latency (F) of hidden platform tests 5, 6, 7, and 8 d post exposure, and representative motion trajectory (G) and platform crossings (H) of probe tests 9 d post exposure are presented. I–K) Barnes maze assays (*n* = 12 mice). Representative motion trajectory (I). Escape latency (J). Number of errors in locating the target (K). L–N) Y‐maze tests (*n* = 12 mice). Heat map of the average motion of each group (L). Novel arm time (%) (M). Novel arm distance (%) (N). O–Q) Novel object exploration experiment (*n* = 12 mice). Heat map of the average motion of each group (O). Total time to explore the novel object (P). Number of times to seek the novel object (Q). All data are presented as mean ± standard error of the mean. Repeated‐measures analysis of variance was performed to analyze the repeated measurement data (B,F,J). Student's *t*‐test was performed to compare the differences between two groups per time point (D,H,K,M,N,P,Q). *, *P* < 0.05; **, *P* < 0.01; N.S., non‐significant (*P* > 0.05).

Memory is the basis and premise of cognition and is easily quantified and detected in animal models. We first assessed the effects of RF exposure on spatial learning and memory using behavioral experiments. The Morris water maze (MWM) is a method designed to assess spatial or place learning and memory ability in rodents, which is strongly correlated with hippocampus (HPC) synaptic plasticity.^[^ ^9^ ^]^ Through hidden platform tests, we found that the average escape latency in RF‐irradiated mice was significantly extended 2 and 3 d post radiation (**Figure** **2**A,B). The platform was then removed and a probe trial was conducted, and the number of platform‐site crossovers in mice of the RFR group showed a decreasing trend at 4 d post radiation, but no statistical difference was identified (Figure 2C,D). Thereafter, the platform was relocated to the opposite quadrant and spatial reversal tests were conducted. We found that the latency increased significantly from 5 to 8 d in RF‐exposed mice (Figure 2E,F), and the number of platform‐site crossovers decreased significantly at 9 d post radiation (Figure 2G,H). These results indicate that RFR causes a decline in spatial learning and memory in mice.

Unlike the MWM, in which animals face great environmental pressure because of their natural aversion to water, the Barnes maze is used to evaluate spatial memory in rodents but brings much less stress to animals.^[^ ^10^ ^]^ Through the Barnes maze test, we found that the escape latency of mice exposed to RFR was significantly extended from 6 h to 14 d post radiation (Figure 2I,J). In addition, the number of errors in locating the target hole increased from 3 to 14 d after RF exposure (Figure 2K). Consistent with the MWM test, the data obtained from the Barnes maze test supported that RFR caused impairment in spatial learning and memory ability in mice.

A Y‐maze – novel arm discrimination experiment was conducted to evaluate spatial recognition memory in mice. In the experiment, probe tests were performed 5 min after memory‐acquisition to assess changes in short‐term memory caused by RFR. Mice in the RFR group showed reduced exploration of the novel arm compared to those in the Con group (Figure 2L,M). Meanwhile, the distance traveled in the novel arm (%) in RFR‐irradiated mice was significantly decreased 6 h after RF exposure (Figure 2N). These results suggest that RFR adversely affects spatial recognition memory in mice.

In addition, a novel object exploration (NOE) test was performed to assess novelty‐seeking behavior in mice. Notably, the total time and number of times the mice explored the novel object in the center of box were significantly reduced in the RFR group 6 h and 7 d after RF treatment (Figure 2O–Q). These findings imply that RF exposure inhibits novelty‐seeking behavior in mice.

Through this part of the study, we found that 2856 MHz RF exposure below the thermal threshold (≤1 °C) had an adverse effect on the HPC‐dependent spatial and place memory ability (both long‐term and short‐term) and novelty‐seeking behavior in mice. In addition, we demonstrated that RF did not affect the motor ability of mice under these conditions (Figure S1A,B, Supporting Information) but tended to cause mild anxiety in mice in an open‐field test (OFT) (Figure S1C,D, Supporting Information). Moreover, no obvious structural damage to the HPC was observed in RF‐exposed mice (Figure S1E, Supporting Information).

### 2.3 RF Exposure Causes No Obvious Change in Local Glutamate Release of Dorsal HPC CA1 in Mice but a Significant Decrease in Dopamine Release

**Figure 3:**
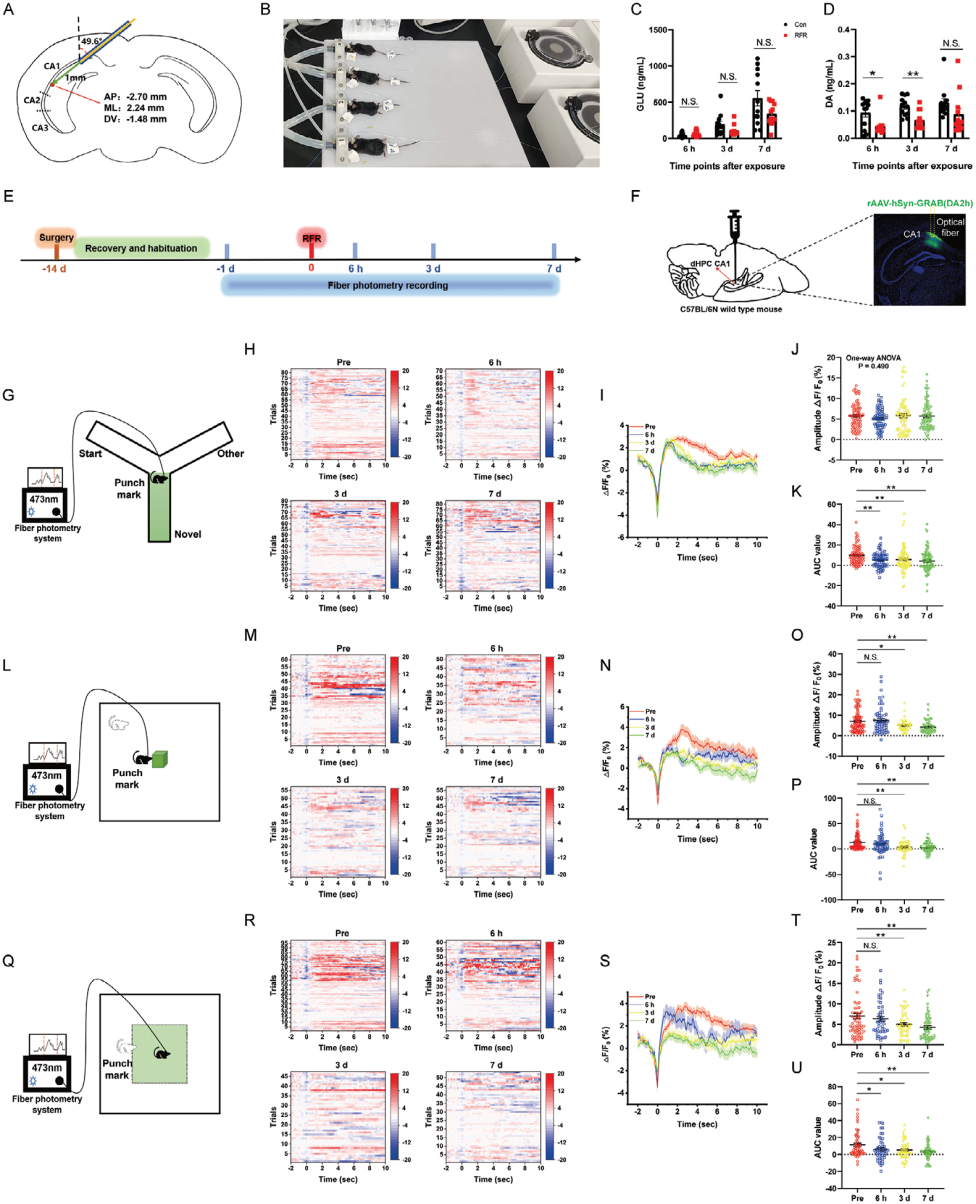
Effects of radiofrequency exposure on the release of glutamate and dopamine in dorsal hippocampus CA1. A–D) Microdialysis sampling and high‐performance liquid chromatography (HPLC) analysis (*n* = 12 mice). Schematic of microdialysis probe location in the dorsal hippocampus (dHPC) CA1. Green represents the membrane of the probe (A). Image of microdialysis sampling in anesthetized mice (B). C,D) The release of glutamate (GLU) and dopamine (DA) detected by HPLC. E–U) Examination of GPCR‐activation‐based‐DA (GRAB_DA_) expressed in dHPC CA1 of mice by fiber photometry. Time course of this experiment. The data collected −1 d pre‐RF exposure (Pre) were used as internal control (E). Schematic of adeno associated virus (AAV) injection and images of GRAB_DA_ expression (F). G–K) GRAB_DA_ signals when mice were performing Y‐maze task (*n* = 7 mice). Schematic of the experimental device. Only GRAB_DA_ signals when mice entered the novel arm were analyzed (G). Heat maps of Δ*F*/*F*_0_ (%) (H). Plot of average Δ*F*/*F*_0_ (%) (I). Plot of amplitude Δ*F*/*F*_0_ (%) (J). Plot of area under the curve (AUC) (K). L–P) GRAB_DA_ signals when mice were performing novel object exploration task (*n* = 7 mice). Schematic of the experimental device. Only GRAB_DA_ signals when mice sought the novel object were analyzed (L). Heat maps of Δ*F*/*F*_0_ (%) (M). Plot of average Δ*F*/*F*_0_ (%) (N). Plot of amplitude Δ*F*/*F*_0_ (%) (O). Plot of AUC (P). Q–U) GRAB_DA_ signals when mice moved freely in an open field (*n* = 7 mice). Schematic of the experimental device. Only GRAB_DA_ signals when mice entered the central area were analyzed (Q). Heat maps of Δ*F*/*F*_0_ (%) (R). Plot of average Δ*F*/*F*_0_ (%) (S). Plot of amplitude Δ*F*/*F*_0_ (%) (T). Plot of AUC (U). Data are expressed as mean ± standard error of the mean. Student's *t*‐test was performed to compare the differences between two groups per time point (C,D). One‐way analysis of variance followed by Bonferroni's correction was used to compare multiple groups (J,K,O,P,T,U). *, *P* < 0.05; **, *P* < 0.01; N.S., non‐significant (*P* > 0.05).

The HPC serves a critical function in memory, navigation, and cognition. HPC CA1 pyramidal neurons (PNs) have place cell activity, of which inhibited long‐term potentiation (LTP) leads to spatial learning and memory impairment.^[^ ^11^ ^]^ Importantly, glutamate (GLU) and dopamine (DA) are neurotransmitters involved in memory coding and consolidation.^[^ ^12^ ^]^ Here, we performed microdialysis sampling of the local dorsal HPC (dHPC) CA1 region in mice under anesthesia and screened for changes in the release of neurotransmitters caused by RF exposure. We found that the release of GLU in dHPC CA1 showed no significant changes in RF‐irradiated mice, whereas the release of DA was significantly downregulated at 6 h and 3 d post radiation (**Figure** **3**A–D). This part of the experiment suggested that the RFR applied in the study caused abnormalities in neurotransmitter release. However, the data were obtained from mice under anesthesia, which may be different from awake mice.

Additionally, genetically encoded GPCR‐activation‐based‐DA (GRAB_DA_) sensors were used to study DA release in the dHPC CA1 area in awake mice.^[^ ^13^ ^]^ The temporal design of this experiment is shown in Figure 3E. The data collected before RF exposure for each experiment were used as internal controls. The expression of GRAB_DA_ in the dHPC CA1 was verified by microscopy at the end of each experiment (Figure 3F). The fluorescence data of GRAB_DA_ were collected using a fiber photometry system when the mice were performing behavioral experiments. Only GRAB_DA_ signals were analyzed when mice entered the novel arm of the Y‐maze (Figure 3G–K), explored the novel object (Figure 3L–P), or entered the center of the open field (Figure 3Q–U). We found that the intensity of DA signal waveforms in the dHPC CA1 was suppressed from 6 h to 7 d after RF exposure in the different behavioral tests, as shown by the reduced amplitude Δ*F*/*F*_0_ (%) and area under the curve (AUC). No change in the fluorescence signal was detected in control mice expressing GFP (Figure S2, Supporting Information). In conclusion, we verified that RFR reduced DA release in the dHPC CA1 in freely moving mice.

### 2.4 RF Exposure Enhances PN (Source of GLU) Activity in dHPC CA1 In Vivo by Nonthermal Mechanisms, Which Recover to Basal Level With RFR Termination

**Figure 4:**
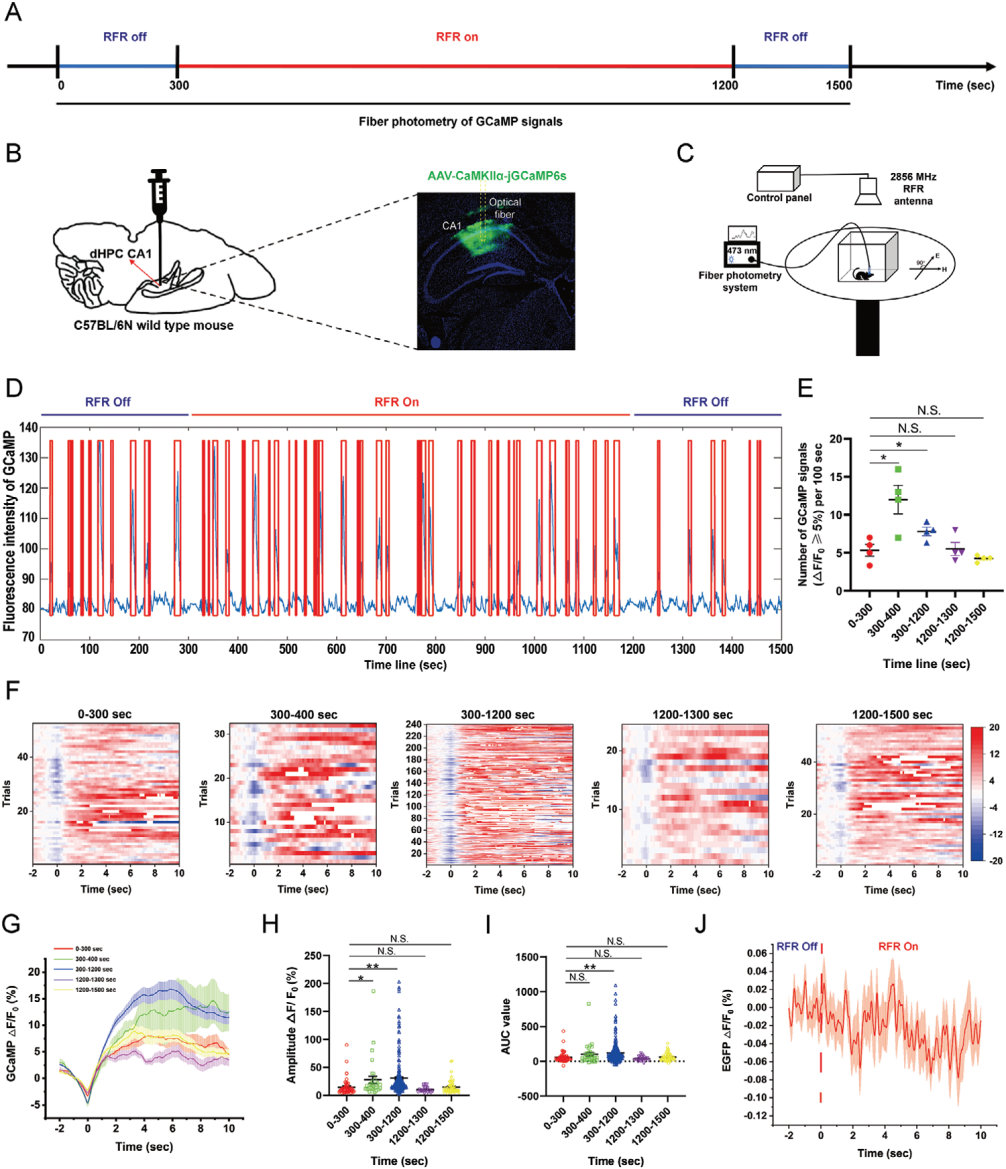
Effects of radiofrequency exposure on the ongoing Ca^2+^ activity of dorsal hippocampus CA1 pyramidal neurons in mice. A) Timeline of the experiment. B–I) Fiber photometry of jGCaMP6s expressed in pyramidal neurons (PNs) of dorsal hippocampus (dHPC) CA1 before, during, and after radiofrequency exposure (*n* = 4 mice). Experimental paradigm of adeno associated virus (AAV) injection and images of jGCaMP6s expression (B). Schematic of the experimental device (C). Representative images of jGCaMP6s fluorescence intensities. The red box indicates jGCaMP6s signals with Δ*F*/*F*_0_ ≥ 5% (D). Statistical analyses of the number of jGCaMP6s signals with Δ*F*/*F*_0_ ≥ 5% per 100 s (E). Heat maps of Δ*F*/*F*_0_ (%) (F). Plot of average Δ*F*/*F*_0_ (%) (G). Plot of amplitude Δ*F*/*F*_0_ (%) (H). Plot of area under the curve (AUC) (I). J) Fiber photometry of green fluorescent protein (GFP) expressed in dHPC CA1 PNs. RFR‐off and RFR‐on showed no impact on GFP fluorescence intensity. Data are expressed as mean ± standard error of the mean. One‐way analysis of variance followed by Bonferroni's post hoc test was used to compare multiple groups (E,H,I). *, *P* < 0.05; **, *P* < 0.01; N.S., non‐significant (*P* > 0.05).

As HPC PNs are responsible for spatial learning and memory,^[^ ^11^ ^]^ we first examined the effects of RFR on HPC PN activity during and after exposure. The temporal design of the experiment is shown in **Figure** **4**A. Adeno associated virus (AAV) expressing jGCaMP6s under the control of the CaMKII*α* promoter was inoculated in CA1 PNs of the dHPC, and the expression of jGCaMP6s was verified by microscopy (Figure 4B). Moreover, the ongoing Ca^2+^ activity of dHPC CA1 PNs was monitored using fiber photometry 300 s before, 900 s during, and 300 s after RF exposure under free exploration of the open‐field box placed directly under the RF antenna (Figure 4C). Spontaneous Ca^2+^ signals (jGCaMP6s) were collected and analyzed. We found that real‐time RF exposure significantly enhanced the activity of CA1 PNs compared with that before RF treatment, showing elevated signal frequencies (Figure 4D,E) and increased signal amplitude Δ*F*/*F*_0_ (%) or AUC (Figure 4F–I), while no effects of RF on GFP‐expressing PNs were identified (Figure 4J).

Interestingly, the activity of PNs in dHPC CA1 was significantly enhanced in the first 100 s after RFR start‐up (time line 300–400 s), while the rectal temperature increased only by 0.1 °C (Figure 1J, Figure 4D–I). Moreover, in the first 100 s after RF termination (time line 1200–1300 s), the state of neurons changed from excitation to a little inhibition (Figure 4H,I, lower amplitude and AUC than the pre‐treatment level, but with no statistical difference), which might be explained by a slight refractory period after a long period of excitement, and the rectal temperature increased by 0.67 °C in relation to the underlying value at this time (Figure 1J). Anyway, neuronal activity recovered to pre‐treatment levels within 300 s after RFR termination (time line 1200–1500 s, Figure 4D–I). No long‐lasting effects of RF exposure on the activity of dHPC CA1 PNs were identified at 6 h, 3 d, and 7 d post RF exposure (Figure S3, Supporting Information). These results indicate that RF exposure enhanced the ongoing Ca^2+^ activities of dHPC CA1 PNs, which seemingly showed no relationship with RF‐induced hyperthermia.

### 2.5 RF Exposure Damages the Locus Coeruleus‐to‐dHPC Dopaminergic Axonal Projection Circuit, Which is the Main Source of DA Release in the dHPC

Numerous studies have demonstrated that DA in the dHPC is mainly derived from locus coeruleus (LC) tyrosine hydroxylase‐expressing (TH^+^) neuronal projections.^[^ ^12a–c^ ^]^ Therefore, studying the impact of RFR on the LC‐to‐dHPC TH^+^ projection loop helps determine the mechanism underlying abnormal DA release in dHPC caused by RF exposure.

**Figure 5:**
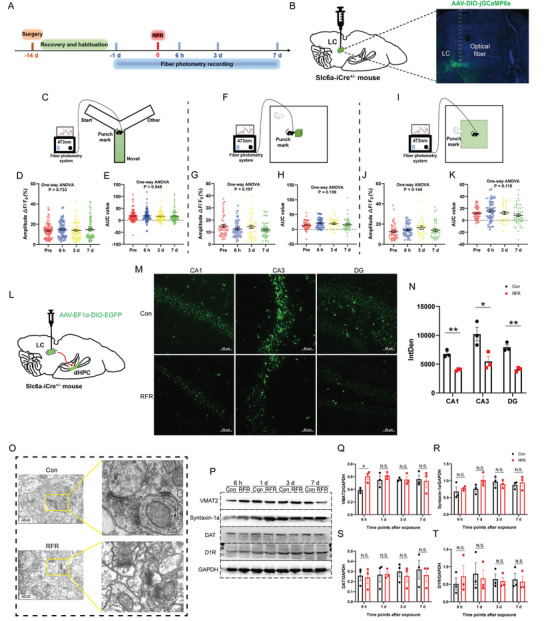
Effects of radiofrequency exposure on the locus coeruleus‐to‐dorsal hippocampus dopaminergic projection. A) Timeline of the experiments. B) Experimental paradigm of adeno associated virus (AAV) injection and images of jGCaMP6s expression. C–K) Fiber photometry on jGCaMP6s expressed in locus coeruleus (LC) dopaminergic (DAergic) neurons in mice. C–E) jGCaMP6s signals when mice performed Y‐maze tests (*n* = 6 mice). Schematic of experimental device (C). Plot of amplitude Δ*F*/*F*_0_ (%) (D). Plot of area under the curve (AUC) (E). F–H) jGCaMP6s signals when mice explored a novel object (*n* = 6 mice). Schematic of experimental device (F). Plot of amplitude Δ*F*/*F*_0_ (%) (G). Plot of AUC (H). I–K) jGCaMP6s signals when mice entered the central of an open field (*n* = 6 mice). Schematic of experimental device (I). Plot of amplitude Δ*F*/*F*_0_ (%) (J). Plot of AUC (K). L–N) Axonal tracing of DAergic projection from the LC to the dorsal hippocampus (dHPC) (*n* = 3 mice). Experimental paradigm of AAV injection (L). Representative images of DAergic axonal terminals in the dHPC CA1, CA3, and dentate gyrus (DG) projected from the LC (Scale bar = 50 µm), detected 6 h after RF exposure (M). Statistical analysis of the density of the DAergic axons in dHPC depicted by integrated optical density (IntDen) (N). O) Synapses in dHPC CA1 observed by transmission electron microscope (Scale bar = 200 nm). P–T) Expression of vesicular monoamine transporter (VMAT2), synaptic fusion protein 1a (syntaxin‐1a), DA transporter (DAT), D1‐receptor (D1R), and glyceraldehyde‐3‐phosphate dehydrogenase (GAPDH) in hippocampus in mice (*n* = 3 mice). Representative images of protein bands by western blotting (P). Statistical analysis results of the expression of VMAT2, syntaxin‐1a, DAT, and D1R normalized by GAPDH (Q–T). Data are presented as mean ± standard error of the mean. One‐way analysis of variance followed by Bonferroni's post hoc test was used to compare multiple groups (D,E,G,H,J,K). Student's *t*‐test was performed to compare the differences between two groups per time point (N,Q–T). *, *P* < 0.05; **, *P* < 0.01; N.S., non‐significant (*P* > 0.05).

First, we explored the effect of RF exposure on LC dopaminergic (DAergic) neuronal activity using fiber photometry on *Slc6a‐iCre* mice infected with AAV‐EF1*α*‐DIO‐ jGCaMP6s in the LC. The timeline of the experiment is illustrated in **Figure** **5**A. The expression of jGCaMP6s in LC DAergic neurons was verified using microscopy (Figure 5B). The jGCaMP6s signals were recorded when mice performed the behavioral tasks, including the Y‐maze, NOE, or OFT. The data collected for pre‐RF exposure in each behavioral experiment were used as controls. No significant differences in the jGCaMP6s signal amplitude Δ*F*/*F*_0_ (%) and AUC were observed at 6 h, 3 d, and 7 d post‐radiation compared with that pre‐radiation, when mice explored the novel arm of the Y‐maze, sought the novel object during NOE, or entered the central area in the OFT (Figure 5C–K). Therefore, we concluded that RFR had no effects on the LC DAergic neuronal activities, which was not a factor resulting in abnormal DA release in the dHPC CA1 in RF‐irradiated mice.

To identify the DAergic projection from the LC to the dHPC, we first selectively labeled the DA axons in the dHPC using the Cre‐Lox method. In brief, mice expressing Cre under the control of the DA transporter (*Slc6a‐iCre*) were used, and DAergic cell bodies in the LC and their axonal projections were transfected by injecting a DIO‐AAV encoding GFP (AAV‐EF1*α*‐DIO‐EGFP) to allow for axonal tracing (Figure 5L). Six hours after RF exposure, we examined the density of LC‐to‐dHPC DAergic projections by integrated optical density (IntDen) and found that the DAergic projections from LC to dHPC were significantly reduced in dHPC CA1, CA3, and dentate gyrus in RF‐irradiated mice (Figure 5M,N). This part of the study showed that LC DAergic neurons started to lose their axonal projections in the dHPC early after RF exposure, which constituted an important mechanism of abnormal DA release caused by RFR.

Molecules expressed in synaptic DAergic terminals, including the vesicular monoamine transporter (VMAT2), synaptic fusion protein 1a (syntaxin‐1a), dopamine transporter (DAT), and D1 receptor (D1R), play important roles in the regulation of DA synaptic vesicle transport, presynaptic exocytosis, reuptake, and postsynaptic activation.^[^ ^14^ ^]^ As structure is the basis of function, we first observed the synapses in dHPC CA1 by transmission electron microscopy and found that the boundary of synaptic structure was not clear, which might be attributed to disordered molecular arrangement (Figure 5O). Additionally, we explored the changes in the molecular mechanisms related to DA synaptic transmission in the HPC of mice by western blotting. We found that only the expression of VMAT2 was transiently upregulated 6 h post radiation, and no effects of RFR on the expression of syntaxin‐1a, DAT, and D1R were identified (Figure 5P–T). These results indicated that the destruction of synaptic structures could be another cause of abnormal DA release in dHPC induced by RF exposure.

### 2.6 Optogenetic Activation of DAergic Terminals or Drug Activation of D1 Receptor in dHPC CA1 Improves RFR‐Caused Spatial Memory Impairment in Mice

**Figure 6:**
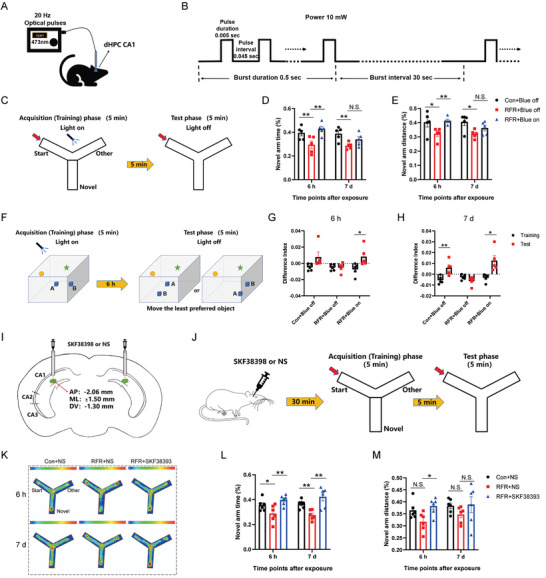
Dopamine activation in dorsal hippocampus CA1 improved spatial learning and memory impairment caused by radiofrequency exposure. A) Schematic of experimental device. B) Optogenetic modulation parameters. C–E) Performance of mice receiving optogenetic stimulation in Y‐maze test. Experiment design (C). Novel arm time (%) (D). Novel arm distance (%) (E). F–H) Performance of mice receiving optogenetic stimulation in spatial object recognition test. Experiment design (F). Difference index of each group 6 h and 7 d post exposure (G,H). I–M) Performance of mice pretreated with SKF38398 or normal saline (NS) in Y‐maze test. Schematic of drug administration (I). Experiment design (J). Heatmap of average movement at 6 h and 7 d post radiation (K). Novel arm time (%) (L). Novel arm distance (%) (M). Data are presented as mean ± standard error of the mean. One‐way analysis of variance followed by Bonferroni's post hoc test was used to compare multiple groups (D,E,L,M). Student's *t*‐test was performed to compare the differences between two groups per time point (G,H). *, *P* < 0.05; **, *P* < 0.01; N.S., non‐significant (*P* > 0.05).

To explore the role of DA in the process of spatial memory dysfunction in mice caused by RF exposure, we examined mouse performance in a Y‐maze task or spatial object recognition task while activating the DA signal in the dHPC CA1 area. *Slc6a‐iCre^+/−^: Rose26‐ChR2^+/−^* model mice were used as the research object, and the ChR2‐expressing DAergic axons in the dHPC CA1 were significantly activated while receiving blue optical stimulation (10 mW, 20 Hz, 5‐ms pulses) (Figure S4, Supporting Information). A Y‐maze experiment was performed to identify spatial learning and memory ability in mice. We allowed the mice to explore the Y‐maze (novel arm closed) during a 5‐min training session. During this session, only mice in “RFR + Blue on” group received intrahippocampal CA1 optical stimulation, while mice in “Con + Blue off” group and “RFR + Blue off” group were connected to the optogenetic system but no optical stimulation was given (**Figure** **6**A–C). Five minutes later, the mice were placed in the Y‐maze (novel arm open) for free exploration for 5 min. We found that, in RFR‐exposed mice, optical activation of ChR2^+^ DAergic axons in the dHPC CA1 during training significantly increased the novel arm time (%) and distance (%) in the test session, which improved the short‐term spatial memory impairment caused by RFR (Figure 6D,E). The same effect was achieved by activating ChR2^+^ DAergic neurons in the LC region (Figure S5A–E, Supporting Information).

A spatial object recognition task was conducted (Figure 6F). We allowed mice to explore a square arena with distinct walls containing two identical objects during a 5‐min training session, during which mice in the “Blue on” group were given optical stimulation while mice in “Blue off” group were not. Six hours later, the mice were challenged to explore the arena during a 5‐min test session, in which the least preferred object was moved to another position. The difference index, a normalized measure indicating the relative amount of time spent exploring the displaced object, was calculated to assess mouse performance.^[^ ^12b^ ^]^ We found that optical activation of dHPC CA1 DAergic axon terminals or LC DAergic neurons during training significantly increased the average difference index during the test session (Figure 6G,H, Figure S5F–H, Supporting Information). These results suggest that photostimulation of the DA signal in dHPC CA1 or LC promotes long‐term spatial memory in RFR‐irradiated mice.

In addition, the DA D1R agonist SKF38398 or an equal amount of normal saline (NS) was locally administered to the bilateral dHPC CA1 region through the pre‐implanted microtubules (Figure 6I). After stabilization for 30 min, we examined performance using the Y‐maze test (Figure 6J). We found that SKF38398 significantly improved spatial learning and memory in mice after RF exposure, whereas NS had no significant effects (Figure 6K–M). Moreover, DA D1R antagonists SCH23390 administrated locally to bilateral hippocampal CA1 could recapitulate the deleterious effects on the spatial recognition memory in mice of RF exposure (Figure S6, Supporting Information). In short, this part of the experiment demonstrated that the decrease in dHPC CA1 DA release was an important cause of long‐term and short‐term HPC‐dependent spatial memory impairment in RFR‐treated mice.

## 3 Discussion

Generally, the biological effects of RF‐EMFs are classified as thermal or nonthermal, based on whether changes in temperature affect the animal's biology.^[^ ^5^ ^]^ However, whether RFR has nonthermal effects and the underlying mechanisms remain controversial. There is increasing evidence that nonthermal RF exposure causes brain damage, including learning and memory impairment,^[^ ^1^ ^]^ sleep disorder,^[^ ^2^ ^]^ anxiety and depression,^[^ ^3^ ^]^ EEG changes,^[^ ^4^ ^]^ and even tumors,^[^ ^5^ ^]^ which cannot be explained by thermal effects. In particular, many laboratories have consistently reported the effect of nonthermal RFR on brain activity measured by EEG.^[^ ^4^ ^]^ However, no definitive evidence of the nonthermal effects of the RFR has been observed. Neuronal activity is the basis of cognitive function and EEG, and there is a clear correspondence between neuronal Ca^2+^ signaling, neuronal activation, and impulse transduction (the physiological basis of EEG).^[^ ^15^ ^]^ In the present study, we explored the effects of RFR on neuronal activity via nonthermal mechanisms. Briefly, we monitored changes in neuronal Ca^2+^ activity and rectal temperature using metal‐free sensors in awake mice exposed to RFR. We found that the 2856 MHz RF applied in this study enhanced the Ca^2+^ activity of the PNs (manifested as increased frequency and amplitude) in dHPC CA1, which showed no relationship with RFR‐induced hyperthermia. In our published studies, we found that 300 kV m^−1^ electromagnetic pulses slightly inhibited ongoing neuronal activity in the dHPC CA1 (with no significant statistical difference).^[^ ^16^ ^]^ Similarly, Yaghmazadeh et al. studied the effect of 950 MHz RF on neuronal activity using a miniature 1‐photon Ca^2+^ imaging device but reported no neuronal responses using metal‐free optical recordings at induced local electric field strengths up to 230 V m^−1^.^[^ ^17^ ^]^ Therefore, we concluded that RF exposure could modulate neuronal activity by nonthermal mechanisms, which are affected by the frequency of the electromagnetic field.

As demonstrated in our studies and other laboratories, nonthermal RF exposure causes damage to brain cognitive function, especially learning and memory, but the underlying mechanisms are yet to be clarified.^[^ ^1^ ^]^ A complex nervous system can be simplified into a model composed of impulse generators (neurons), connecting lines (neural circuits), and nodes (synapses). Among these, synapse plasticity, LTP in particular, is the most intensively studied cellular model of the memory.^[^ ^18^ ^]^ Synapses are the nodes of neural circuits, and abnormalities in synaptic structural and functional plasticity may contribute to cognitive injury caused by RFR.^[^ ^19^ ^]^ Previous studies have reported that abnormal synaptic plasticity, such as NMDAR‐mediated LTP, is an important mechanism of RFR‐induced cognitive impairment.^[^ ^1^, ^20^ ^]^ In the work presented here, we established an animal model of HPC‐dependent spatial learning and memory impairment by exposing mice to 2856 MHz RFR within the range of body thermal noise (≤1 °C). LC‐to‐dHPC DAergic projections are required for the persistence of synaptic plasticity and memory, thereby playing an important role in novelty‐associated memory enhancement.^[^ ^12a–c^ ^]^ We found that RF exposure caused a significant decline in DA release in the dHPC CA1, resulting in impairment of HPC‐dependent spatial learning and memory in mice. We then examined the effects of RF exposure on the LC‐to‐dHPC DAergic projection loop and clarified that the damaged neural circuit was responsible for the reduced DA release and memory dysfunction induced by RF exposure. Therefore, in addition to synaptic plasticity, damage to neural circuits is an important mechanism underlying brain injury due to RF exposure.

**Figure 7:**
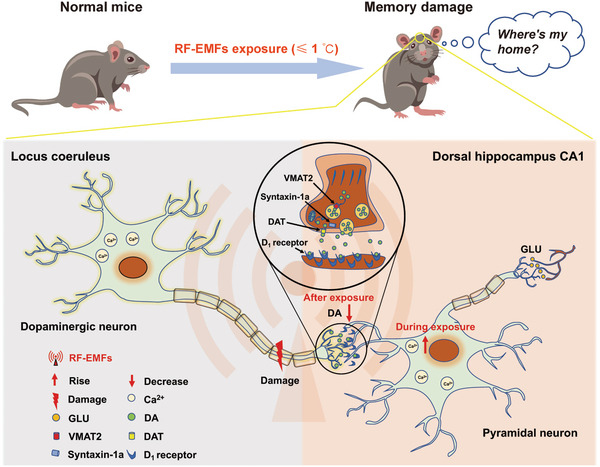
Effects of radiofrequency exposure on neuronal activity and neural circuit loop. Radiofrequency (RF) stimulation applied in the study enhances the ongoing activity of pyramidal neurons (PNs) in the dorsal hippocampus (dHPC) CA1 in mice by a nonthermal mechanism, which recovers to the basal level with the termination of RF exposure. In addition, RF exposure under this condition damages the locus coeruleus‐to‐dorsal hippocampus dopaminergic axonal projection circuit, reduces the release of dopamine in dHPC CA1, and impairs hippocampus‐dependent spatial learning and memory in mice. RF‐EMFs: radiofrequency‐electromagnetic fields; GLU: glutamate; DA: dopamine; VMAT2: vesicular monoamine transporter 2; DAT: dopamine transporter; Syntaxin‐1a: synaptic fusion protein 1a.

In conclusion, our study demonstrated that RF exposure could modulate ongoing neuronal activity in vivo through nonthermal mechanisms. However, the mechanism underlying the effect of nonthermal RF on neuronal activity needs to be further explored, and its relationship with the frequency and modulation of RF‐EMFs is yet to be clarified. In addition, we identified that long‐distance neural circuits are potential targets sensitive to RFR damage (**Figure** **7**).

## 4 Experimental Section

### Animals

All experimental procedures were approved by the Institutional Animal Care and Use Committee of the Beijing Institute of Radiation Medicine (No. IACUC‐DWZX‐2021‐570). *Slc6a (DAT)‐iCre^+/−^* male mice, *Rose26‐ChR2^+/−^* male mice on a C57BL/6N background (BIOCYTOGEN Technology Co., Ltd., Beijing, China), and naive C57BL/6N male mice (Beijing Vital River Laboratory Animal Technology Co., Ltd., Beijing, China) were used for this study. *Slc6a‐iCre^+/−^: Rose26‐ChR2^+/−^* male mice were generated by crossbreeding. All mice were housed under a 12‐h light/dark cycle at 18–22 °C and relative humidity 50–60% with free access to food and water. They were randomly divided into a Con group and RFR group. At the start of the trial, all the mice were 8‐weeks‐old.

### RF Exposure System

The RF exposure system used in this study has been described in detail previously.^[^ ^1c^ ^]^ Briefly, the mice in the RFR group were placed in a metal‐free square container made of porous plexiglass. The container was placed on a circular platform directly below the RF horn antenna. Square wave pulses of 2856‐MHz RFR were applied for 900 s, with a peak power density 200 mW cm^−2^, repetition frequency 80 Hz, and duty Cycle 4%. Mice in the Con group were processed in parallel with those in the RFR group, but the RF source was switched off.

### Fiber Optic Thermometer

The rectal temperature of the mice was monitored in real time during RF exposure using a fiber optic thermometer (model THR‐NC‐1084C, FISO, CA). Briefly, mice were raised in cages without padding or other edible items. The solid feed was replaced with 10% sucrose water solution 7 d before the treatment to eliminate the influence of feces in the rectum on temperature detection. Mice were then fixed in a special holder made of acrylic material, placed on the RF exposure platform, and a metal‐free fiber optic thermometer sensor (2 mm diameter) was inserted into the anus with a depth of ≈7 mm. Temperature data were transmitted through the optical fiber and recorded before, during, and after RF exposure.

### Virus Injection and Optical Fiber Implantation

The viruses used in this study included AAV‐CaMKII*α*‐jGCaMP6s, AAV‐EF1*α*‐DIO‐jGCaMP6s, pAAV‐CaMKII*α*‐EGFP‐3xFLAG‐WPRE (OBiO Technology Corp., Ltd., Shanghai, China), and rAAV‐hSyn‐GRAB(DA2h) (Brain Case Biotechnology Corp., Ltd., Shenzhen, China). The viruses were aliquoted and stored at −80 °C. Mice were anesthetized by inhalation of isoflurane at ≈4–5% in the induction phase and 1% during maintenance. Afterward, the head of each mouse was fixed to a brain stereotaxic apparatus (RWD Life Science, China). The eyes were protected by erythromycin eye ointment covering. A heating pad was used to maintain body temperature at 37 °C. After skin preparation and disinfection, the scalp was cut to expose the skull. A small hole was bored in the skull using a mini drill (Strong204, Saeshin, Korea). Viruses (200 nL) were injected into the target brain regions through a glass microneedle at a rate of 30 nL min^−1^ using a microsyringe pump (KDS LEGATO 130, RWD Life Science), after which the needle was left in place for another 10 min. For detecting the release of DA, rAAV‐hSyn‐GRAB(DA2h) (2.40 × 10^12^ viral genomes [vg] mL^−1^) was injected into the dHPC CA1 (AP: −2.06 mm, ML:1.5, DV: −1.3 mm) of wild‐type mice. To detect the activity of dHPC CA1 PNs, AAV‐CaMKII*α*‐jGCaMP6s (6.54 × 10^12^ vg mL^−1^) was injected into the dHPC CA1 of wild‐type mice. For detecting the activity of DAergic neurons in the LC, AAV‐EF1*α*‐DIO‐jGCaMP6s (1.12 × 10^12^ vg mL^−1^) was injected into the LC (AP: −5.40 mm; ML: 0.875 mm; DV: −3.75 mm) of *Slc6a‐iCre^+/−^* mice. pAAV‐CaMKII*α*‐EGFP‐3xFLAG‐WPRE (2.15 × 10^13^ vg mL^−1^) was also injected for control in fiber optic photometry experiments to address possible optical artifacts. An optical fiber (200 µm outer diameter, 0.37 numerical aperture) bound to a ceramic ferrule was implanted with its tip targeting the virus injection site and secured to the skull using dental acrylic. Behavioral tests were performed after 2–3 weeks of recovery and acclimation. After experiments, histological analysis was performed to verify the location of viral infection.

### Behavioral Tests

The operator placed the mice in the behavioral room for 30 min every day for three consecutive days. This habituation procedure was followed for all behavioral experiments. ANY‐maze software (Version 6.32, Stoelting, USA) was used to record and analyze the movements of the mice.

### MWM

The MWM method was performed as described previously.^[^ ^16^ ^]^ Briefly, a water pool with a diameter of 128 cm was used, which was equally divided into four quadrants. A platform (6 cm diameter) was placed in the center of one quadrant, and submerged 1 cm below the water surface. The pool was covered with a light‐colored curtain with different spatial markers on the pool walls. Training was performed before RF exposure once daily for 3 d. During this phase, the mice had 60 s to find the hidden platform, and if they did not find it within this time, they were directed to the platform and allowed to stay on it for another 10 s to encode spatial cues around. Each mouse was tested four times a day, with the starting quadrant randomly selected and without duplication. The hidden platform tests were performed from 6 h to 3 d post radiation, with the platform kept in the same quadrant. The mice were challenged from each of four quadrants within 60 s, and the latency was recorded as 60 s if the mice failed to escape. A probe trial was performed 4 d post radiation, during which mice were placed in the opposite quadrant and allowed to swim freely for 60 s after the platform was removed. Next, the platform was moved to the opposite quadrant and reversal spatial learning experiments were performed, including hidden platform tests from 5 to 8 d post RF exposure and probe trials 9 d post radiation, with the same procedure as above.

### Barnes Maze

The circular platform was 92 cm in diameter and 84 cm above ground. Around the platform were 20 equidistant holes with diameters of 5 cm, one of which was connected to a dark box and designated as the target hole, whereas the other holes were fake. Training was performed before RF exposure once daily for 3 d. First, mice were placed in the center of the platform and restricted for 5 s using a plastic bucket. Subsequently, the bucket was removed and mice were allowed to explore the maze freely. Successful escape was attributed to the mouse entering the target hole with all limbs within 4 min and continuing to stay in it for another 30 s. Otherwise, the mice were guided to the target hole and kept there for 30 s. Thereafter, the probe tests were conducted. Mice were allowed to explore the maze freely for 4 min. If the mice did not enter the target hole within this period, the test was terminated and the escape latency was recorded as 4 min.

### Y‐Maze

The Y‐maze was composed of three identical arms at 120° angles, serving as the start, other, and novel arms. Each arm was 30 cm long, 8 cm wide, and 15 cm high. The interior walls of each arm were adorned with spatial identifiers of various shapes. The novel arm was closed using a baffle during the memory‐acquisition phase. Mice were placed in the Y‐maze from the start arm and allowed to move freely for 5 min. Five minutes later, the test phase began, with all the arms remaining open. The mice were placed in the maze from the start arm and allowed to explore freely for 5 min.

### NOE

The novelty‐seeking behavior of mice was assessed using a NOE experiment. During the test phase, mice were first placed into an open‐field box (40 cm side length) to move freely for 5 min. Subsequently, a novel object was placed in the center of the box, and mice were allowed to explore freely for another 5 min.

### OFT

An open field was used to assess the locomotor and behavioral activity levels of mice treated and not treated with RFR. The open‐field box was 40 cm in length and 35 cm in height, with a central area of 20 × 20 cm, and the remaining space was denoted as the peripheral area. Before formal testing, the environmental acclimation procedure was strictly implemented. During the test phase, the mice were placed in the central area with their backs to the experimenter from a fixed position and were allowed to move freely for 5 min.

### Pathological Examination

The mice were anesthetized with 80 mg kg^−1^ pentobarbital sodium (intraperitoneal injection) and decapitated. HPC tissue was peeled off on ice from the left hemisphere of the brain and fixed in 10% buffered formalin solution, followed by sectioning and hematoxylin and eosin staining. Observations were performed under an optical microscope (Leica, Germany). Likewise, HPC was peeled off from the right hemisphere of the brain, and 1 mm^3^ tissue blocks were fixed with 2.5% glutaraldehyde, followed by embedding, ultrathin sectioning, heavy metal staining, and observation by transmission electron microscopy (Hitachi, Japan).

### Microdialysis Sampling and High‐Performance Liquid Chromatography

Similar to the optical fiber implantation surgery, wild‐type mice were anesthetized by isoflurane inhalation, and the head was mounted onto a brain stereotaxic instrument. The drilling point was marked 0.5 mm horizontally to the left of the fontanelle point, and a hole was made with a mini drill. A metal‐free guide cannula (CMA 7; CMA Microdialysis AB, Sweden) was implanted into the CA1 region of the left HPC with an insertion depth of 2.28 mm at an angle of 49.6° from the vertical direction. The tip of the cannula was targeted in the dHPC CA1 (AP: −2.70 mm, ML:2.24 mm, DV: −1.48 mm) and fixed to the skull using dental acrylic. After 7 d of recovery, further tests were conducted. Microdialysis sampling of the dHPC CA1 region was then performed. Briefly, mice were anesthetized by isoflurane inhalation. The plug in the cannula was removed, and a microdialysis probe (CMA 7; membrane length, 1 mm; outer diameter, 0.24 mm; cut‐off, 6000 Dalton) was inserted. Ringer's solution was pumped from the inlet of the microdialysis probe at a flow rate 0.5 µL min^−1^ using a micropump (CMA 402, CMA Microdialysis AB), and the dialysate was collected with a refrigerated fraction collector (MAB 85, MICRODIALYSIS AB, Sweden). The effluent for the first 30 min after microdialysis start was discarded, and a total of 30 µL dialysate was collected over the next 60 min and stored at −80 °C for further analysis. Notably, for the samples used to detect DA, the collection tube was prefilled with 3 µL of 0.1% perchloric acid. The concentrations of transmitters, including GLU and DA, were detected by high‐performance liquid chromatography (HPLC, Agilent, USA).

### Fiber Photometry

The activity of Ca^2+^ signals in neurons was investigated using a multichannel fiber photometry recording system (Thinker Tech Nanjing Bioscience Inc., Nanjing, China).^[^ ^21^ ^]^ Virus‐infected mice were first habituated to a ceramic ferrule implanted in their brains. Next, an optical fiber was connected to the ferrule. Fiber photometry of calcium signals was performed by transmitting a 473‐nm laser beam through the optical fiber to excite the genetically coded jGCaMP6s or GRAB_DA_ probes. The fluorescence signals generated by the excited jGCaMP6s or GRAB_DA_ were collected using a fiber photometry system. Meanwhile, photometry was performed on GFP‐expressing animals to exclude the influence of possible behavioral artifact(s) and even the effects of RFR on the fluorescent protein itself.^[^ ^22^ ^]^ The power of the excitation light was less than 30 µW to minimize bleaching. The sampling frequency was set at 50 Hz.

Calcium activity in dHPC CA1 PNs was recorded under real‐time RFR conditions. During the tests, the mice were placed in a 40 × 40 cm metal‐free open‐field box directly below the RF antennae and allowed to move freely. jGCaMP6s signals were recorded 300 s before, 900 s during, and 300 s after RFR treatment. MATLAB 2016a software (MathWorks, Cambridge, United Kingdom) was used to analyze photometry data. All spontaneous fluorescent signals were analyzed, and no data were excluded. Baseline correction was performed according to the method described previously.^[^ ^23^ ^]^ The minimum value of the rising edge of the signal waveform was uniformly defined as the starting point of the fluorescence signals (*T* = 0 s). The fluorescence signal changes were derived by calculating Δ*F*/*F*_0_ = (*F*_signal_−*F*_0_)/*F*_0_, where *F*_signal_ was the test fluorescence signal and *F*_0_ was the baseline averaged over a 2.0 s long control time window (−2 to 0 s of Δ*F*/*F*_0_). Δ*F*/*F*_0_ values are presented as heatmaps and average plots. Amplitude was identified as the peak Δ*F*/*F*_0_ of the fluorescence signal waveforms. The frequency of fluorescence signals with Δ*F*/*F*_0_ ≥ 5% was determined. The AUC between Δ*F*/*F*_0_ at 0 s and at the subsequent 10 s was calculated to verify the activation or inhibition intensity of the fluorescence signals.

The effects of RFR on GRAB_DA_ expressed in the dHPC CA1 or jGCaMP6s expressed in the dHPC CA1 PNs and LC DAergic neurons in virus‐infected mice were also studied. GRAB_DA_ or jGCaMP6s signals were collected during mice behavioral tasks, including Y‐maze, NOE, and OFT. The effects of RFR on neuronal Ca^2+^ activities (jGCaMP6s) and neurotransmitters release (GRAB_DA_) were focused on; behavior experiments were performed to control the interference of environmental variables. Therefore, GRAB_DA_ or jGCaMP6s signals were analyzed only when mice explored the novel arm in the Y‐maze, contacted the novel object in NOE, or entered the central area of the open‐field box. The minimum value of the rising edge of the signal waveform was uniformly defined as the starting point of the fluorescence signals (*T* = 0 s). Δ*F*/*F*_0_ were presented as heatmaps and average plots, and amplitude and AUC (0–10 s of Δ*F*/*F*_0_) were also calculated.

### Neuronal Circuit Tracing

AAV‐EF1*α*‐DIO‐EGFP (1.02 × 10^13^ vg mL^−1^, 80 nL total volume), provided by OBiO Technology Corp., Ltd., was injected into the LC area of *Slc6a‐iCre^+/−^* mice, to measure anterograde monosynaptic tracer output to the DAergic axonal terminals in the dHPC. The mice were allowed to recover for 3 weeks to allow full expression of the viruses. Six hours after RF exposure, mice were anesthetized with 80 mg kg^−1^ pentobarbital sodium and decapitated. The brain was completely peeled out. Coronal slices with a thickness of 20 µm were cut using a cryotome (CM 1950, Leica, Germany). Sections containing the dHPC were selected, stained with 4',6‐diamidino‐2‐phenylindole (DAPI), and observed using laser confocal microscope (Leica).

### Western Blotting

At designated time points post radiation, mice were anesthetized using sodium pentobarbital (80 mg kg^−1^) and decapitated. HPC tissue was stripped on ice, and total proteins were extracted. Target proteins were measured with the following antibodies: rabbit anti‐VMAT2 (abcam259970; 1:1000), rabbit anti‐syntaxin‐1a (abcam272736; 1:1000), rabbit anti‐DAT (abcam184451; 1:1000), rabbit anti‐D1R (abcam279713; 1:1000), and mouse anti‐glyceraldehyde‐3‐phosphate dehydrogenase (GAPDH; abcam8245; 1:10000). The membrane was then incubated with the appropriate horseradish peroxidase‐coupled secondary antibody (1:5000) for 1 h at room temperature. The enhanced chemiluminescence reaction was performed and bands were imaged and quantified with Image J (National Institutes of Health, USA).

### Electrophysiological Recordings

In vivo photoelectrode multichannel electrophysiological recording of CA1 neuronal activity in *Slc6a‐iCre^+/−^: Rose26‐ChR2^+/−^* mice expressing ChR2 in DAergic neurons was performed. The optoelectrode consisted of an optical fiber (diameter: 200 µm) surrounded by a 16‐channel unit electrode array comprising 16 insulated nichrome wires (30 µm diameter, 100–250 kΩ). The mice were fixed on a brain stereotaxic apparatus, the skull surface was exposed, a square cranial window (1.5 × 1.5 mm) was opened, and the electrode was slowly advanced into dHPC CA1 (AP: −2.06 mm; ML: +1.50 mm; DV: −1.30 mm). The reference and ground electrodes were small stainless‐steel screws fixed to the skull above the cerebellum using dental cement. The next experiment was performed after 1 week of recovery. The electrodes were connected to the 16‐channel front‐end via an operational amplifier to minimize cable movement artifacts. The amplifier (Blackrock, USA) was connected to a CerePlex Direct system (Blackrock), and the signal was digitized at 40 kHz using Central Suite software (version 7.0.6) and bandpass filtered from 250 Hz to 5 kHz for spike analysis. After neuronal firing activity was stabilized, data collection began; no optical stimulation was given in the first 20 s, and blue light stimulation (473 nm, 10 mW, 20 Hz, duty cycle 10%) was given in the second 20 s by an optogenetic system (Thinker Tech Nanjing Bioscience Inc.). The collected data were analyzed for changes in neuronal firing using NeuroExplorer (version 5.0) software combined with encoding of the given light stimulus.

### Optogenetic Experiments

*Slc6a‐iCre^+/−^: Rose26‐ChR2^+/−^* male mice were implanted with ceramic inserts in the LC through surgery. The mice were randomly divided into three groups: Con group + no blue light stimulation (Con + Blue off), RFR group + no blue light stimulation (RFR + Blue off), and RFR group + blue light stimulation (RFR + Blue on). Daily fiber optic adaptation training was performed at a fixed time. After attaching the optical fiber to the ceramic insert in the head of the mouse, it was placed in a square open‐field chamber for 5 min without blue light stimulation for 3 d.

### Y‐Maze

First, ceramic inserts in mouse head were connected to the blue light stimulation system. The novel arm was closed using a baffle during the memory‐acquisition phase. Mice were placed in the Y‐maze from the start arm and allowed to explore freely for 5 min. During this stage, the “RFR + Blue on” group received blue light stimulation throughout, whereas the mice in the “Con + Blue off” and “RFR + Blue off” groups did not. Five minutes later, the test phase began with all arms remaining open. The fiber optic connection cable was removed from the mice's head, and the mice were reinserted into the Y‐maze from the start arm and allowed to explore freely for 5 min.

### Spatial Object Recognition Experiment

After habituation, the ceramic inserts in the mouse heads were connected to the blue optogenetic system. The mice were placed in the open‐field box (two identical cylinders were placed in two adjacent corners of the open‐field box, 10 cm from the adjacent wall) and allowed to explore for 5 min. During this stage, the “RFR + Blue on” group received blue light stimulation throughout, whereas the mice in the “Con + Blue off” and “RFR + Blue off” groups did not. Six hours later, the fiber optic connection cable was removed from the mouse's head, and the mice were reinserted into the same environment and allowed to explore the chamber during a 5‐min test session, in which the least preferred object (the object that the mice spent the least time exploring during the memory‐acquisition phase) was moved to a different location. ANY‐maze software was used to record the time spent by the mice exploring each object. Difference index = (displaced object time–stationary object time)/total exploration time.

### Drug Administration

Wild‐type male mice were used, and a guide cannula was implanted in the bilateral dHPC CA1 region (AP: −2.06 mm, ML: ±1.5 mm, DV: −1.3 mm) through surgery. The mice were randomly divided into three groups: Con group treated with NS (Con + NS), RFR group treated with NS (RFR + NS), and RFR group treated with drug (RFR + drug). After a week of recovery, further experiments were conducted. SKF38393 (D1 receptor agonist, Sigma–Aldrich, USA) and SCH23390 (D1 receptor antagonist, Sigma–Aldrich) were dissolved in NS to a final concentration of 0.125 and 0.25 µg µL^−1^, respectively. With the help of a microinjector (CMA 402, CMA Microdialysis AB), 0.4 µL of SKF38393 or SCH23390 work solution was injected into the bilateral dHPC CA1 regions in mice of “RFR + drug” group at a rate of 0.2 µL min^−1^, and the needle was left in place for another 5 min. The mice of the “Con + NS” and “RFR + NS” groups only received the same volume of NS at the same coordinates. Y‐maze and spatial object recognition experiments were subsequently performed to evaluate the spatial recognition memory of mice.

### Statistical Analyses

Data from the Con and RFR groups were expressed as the mean ± standard error of the mean (SEM). Student's *t*‐test was used to compare differences between these two groups, one‐way analysis of variance (ANOVA) followed by Bonferroni's post hoc test was used for multiple group comparisons, and repeated‐measures data were analyzed by repeated‐measures ANOVA (RM ANOVA). All statistical analyses were performed using SPSS software (version 20.0; IBM, Armonk, NY, USA). Statistical significance was set at *P* < 0.05.

## Conflict of Interest

The authors declare no conflict of interest.

## Author Contributions

*Conceptualization*: Y.H.H., Y.L., H.Y. *Writing‐Reviewing and Editing*: Y.L. *Methodology, Data Curation*: Y.H.H., W.Q.L., Y.J.L., Y.L., Z.T.X., Y.M.Y., H.M.Z., H.D., H.Y.Z. *Writing‐Original draft preparation*: Y.H.H., W.Q.L. Y.H.H. and W.Q.L. contributed equally to this work. All authors have read and approved the manuscript.

## Supporting information

Supporting Information

## Data Availability

The data that support the findings of this study are available from the corresponding author upon reasonable request.
