# Supplementary material for: Effects of Nonthermal Radiofrequency Stimulation on Neuronal Activity and Neural Circuit in Mice
Source: Adv Sci (Weinh). 2023 Feb 8;10(11):2205988. doi: 10.1002/advs.202205988 (PMC10104648; doi:10.1002/advs.202205988)
Supplement: Supplementary file 1 — Supporting Information [file ADVS-10-2205988-s001.pdf]

ADVANCED SCIENCE
Open Access

Supporting Information

for *Adv. Sci.*, DOI 10.1002/advs.202205988

Effects of Nonthermal Radiofrequency Stimulation on Neuronal Activity and Neural Circuit in Mice

Yanhui Hao, Weiqi Liu, Yujie Liu, Ying Liu, Zhengtao Xu, Yumeng Ye, Hongmei Zhou, Hua Deng, Hongyan Zuo, Hong Yang and Yang Li**

Supplementary Figures

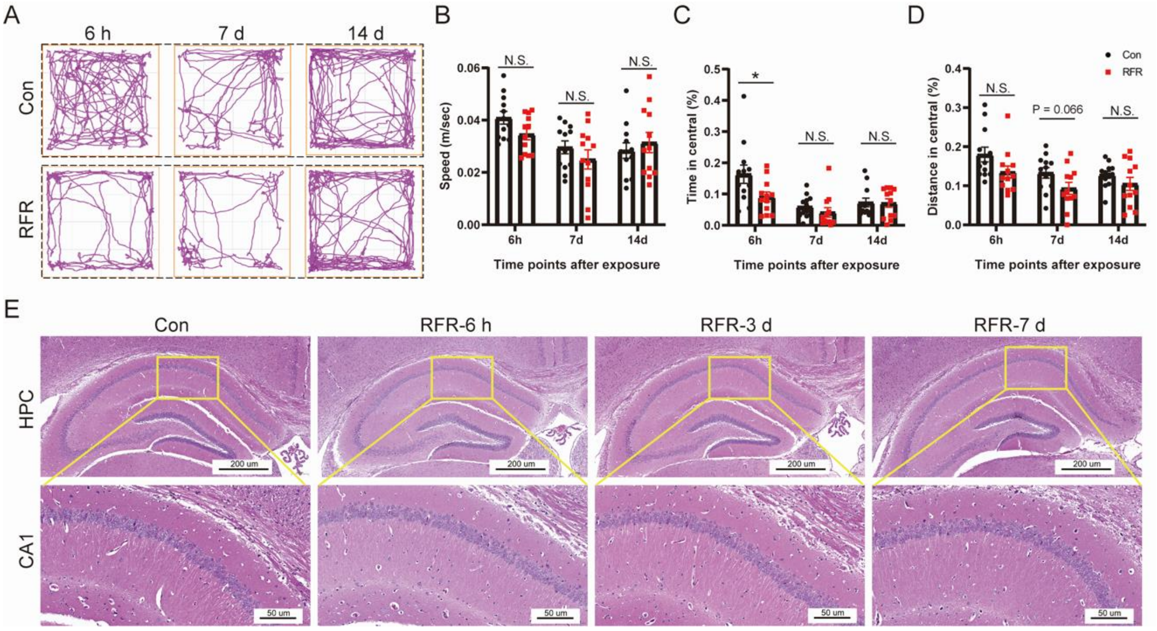

Suppl. Fig. 1 Effects of radiofrequency exposure on the anxiety state and tissue structure of the mouse hippocampus. (A-D) Open field assay. (A) Representative motion trajectory. (B) Speed. (C) Central area time (%). (D) Central area distance (%). **(E)** Tissue structure in dorsal hippocampus (dHPC) stained with hematoxylin and eosin (Scale bar = 50 μm). The yellow square indicates the CA1 area in the dHPC, and partially enlarged images are shown below.

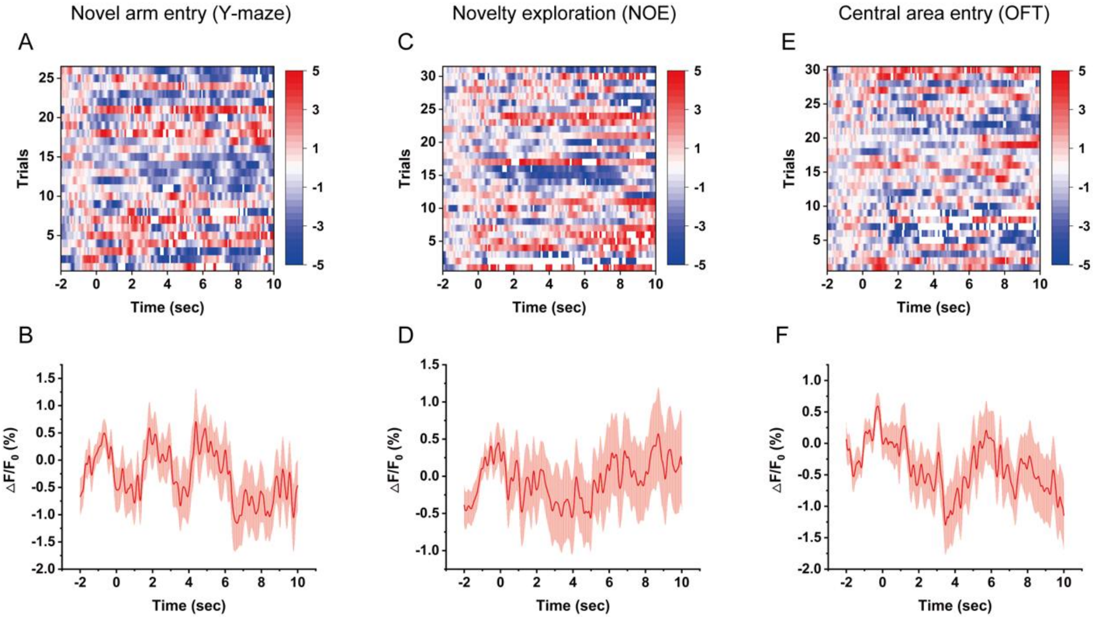

Suppl. Fig. 2 Fiber photometry findings of green fluorescent protein-expressing

mice. (A-B) Green fluorescent protein (GFP) intensity when mice entered the novel arm in a Y-maze novel arm discrimination experiment (0 s for $\Delta F/F_0$), (A) Heat maps of $\Delta F/F_0$ (%) in multiple trials for three mice, (B) Plot of average $\Delta F/F_0$ (%) as shown in A. **(C-D)** GFP intensity when mice sought the novel object in a novel object exploration (NOE) task (0 s for $\Delta F/F_0$), (C) Heat maps of $\Delta F/F_0$ (%) in multiple trials, (D) Plot of average $\Delta F/F_0$ (%) as shown in C. **(E-F)** GFP intensity when mice entered the central area in an open field test (OFT) (0 s for $\Delta F/F_0$), (E) Heat maps of $\Delta F/F_0$ (%) from multiple trials for three mice, (F) Plot of average $\Delta F/F_0$ (%) as shown in E. The red shadow indicates the standard error of the mean.

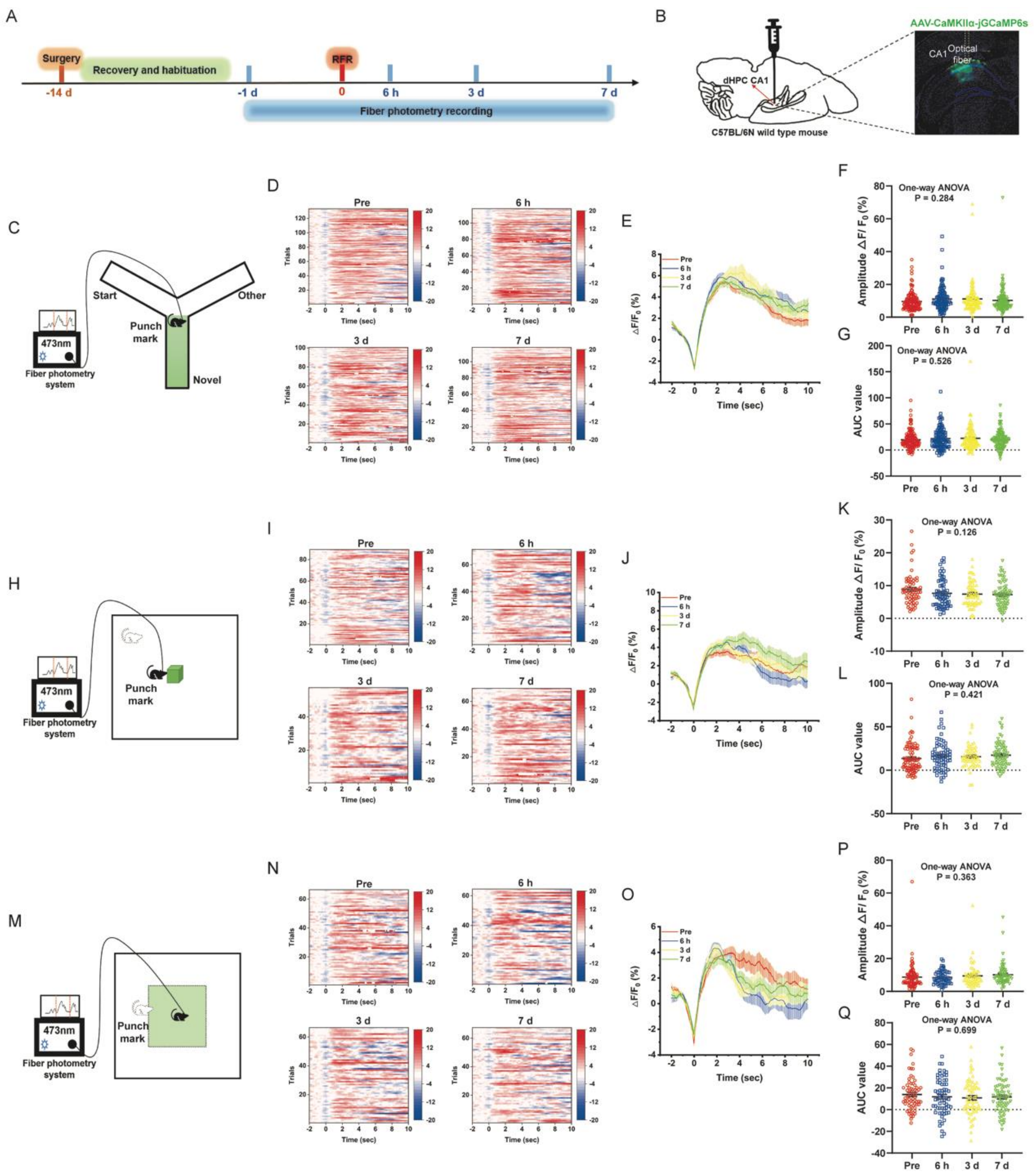

Suppl. Fig. 3 Effects of radiofrequency exposure on the activity of dorsal hippocampus CA1 pyramidal neurons post exposure. (A) Temporal design for this experiment. **(B)** Experimental paradigm for adeno associated virus (AAV) injection and images of jGCaMP6s expression. **(C-G)** Fiber photometry of jGCaMP6s-expressing mice when performing Y-maze test (n = 6 mice). (C) Schematic of the experimental device. Only jGCaMP6s signals noted when mice entered the novel arm were analyzed. (D) Heat maps of $\Delta F/F_0$ (%). (E) Plot of average $\Delta F/F_0$ (%). (F) Plot of amplitude $\Delta F/F_0$ (%). (G) Plot of area under the curve (AUC). **(H-L)** Fiber photometry findings of jGCaMP6s-expressing mice during novel object exploration task (n = 6 mice). (H) Schematic of the experimental device. Only jGCaMP6s signals noted when mice sought the novelty were analyzed. (I) Heat maps of $\Delta F/F_0$ (%). (J) Plot of average $\Delta F/F_0$ (%). (K) Plot of amplitude $\Delta F/F_0$ (%). (L) Plot of AUC. **(M-Q)** Fiber photometry findings of jGCaMP6s-expressing mice moving freely in an open field (n = 4 mice). (M) Schematic of the experimental device. Only jGCaMP6s signals noted when mice entered the central area were analyzed (0 s for $\Delta F/F_0$). (N) Heat maps of $\Delta F/F_0$ (%). (O) Plot of average $\Delta F/F_0$ (%). (P) Plot of amplitude $\Delta F/F_0$ (%). (Q) Plot of AUC. Data are expressed as mean ± standard error of the mean. One-way analysis of variance followed by Bonferroni's post hoc test was performed to compare multiple groups (F-G, K-L, P-Q). *, $P < 0.05$; **, $P < 0.01$; N.S., non-significant ($P > 0.05$).

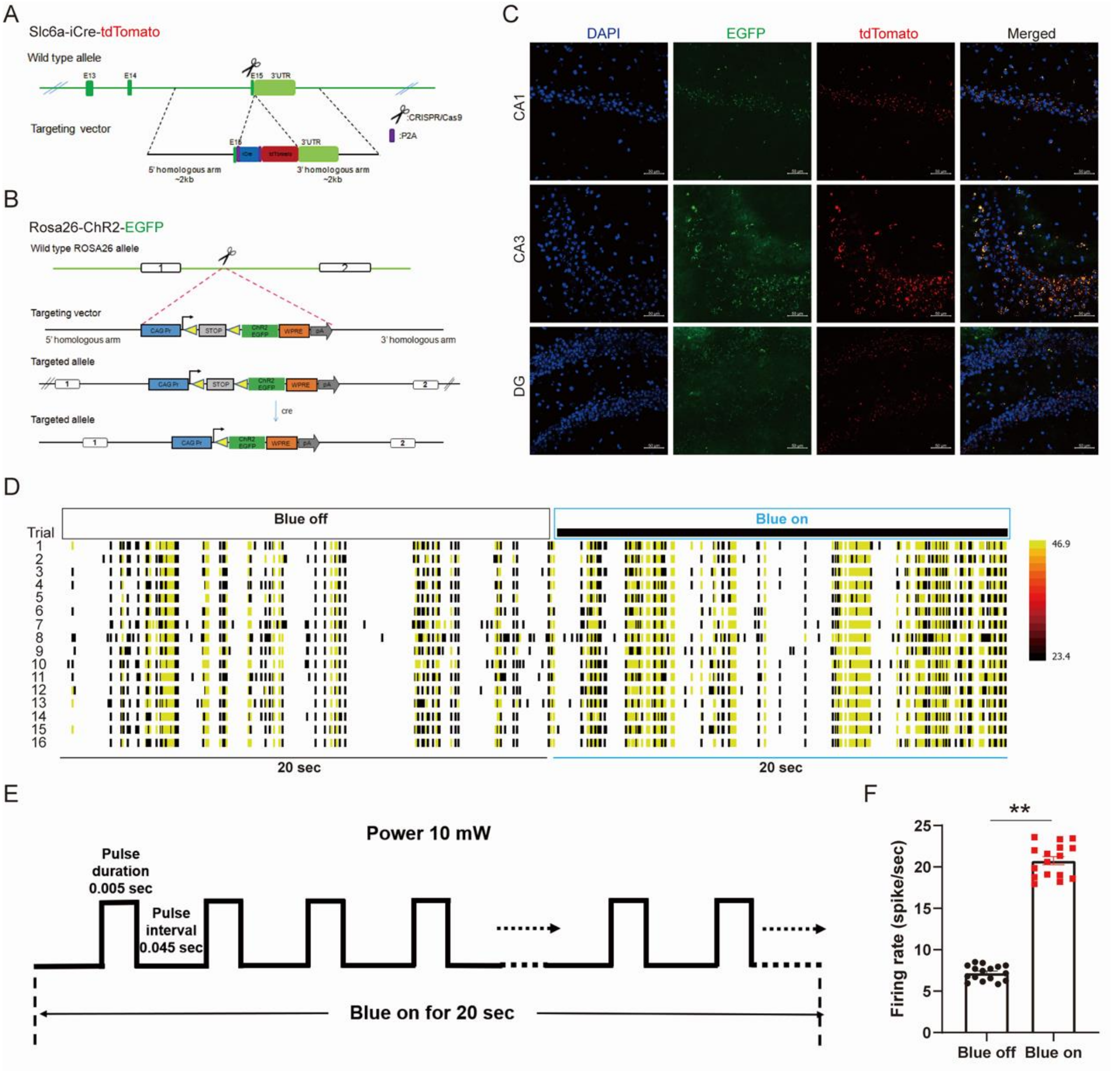

Suppl. Fig. 4 Construction and validation of Slc6a (DA)-iCre and Rosa26-ChR2 model mice. (A-B) Schematic of gene editing strategy. (C) Images of the dorsal hippocampus (dHPC) CA1, CA3, and dentate gyrus (DG) area of Slc6a-iCre$^{+/-}$: Rosa26-ChR2$^{+/-}$ mice obtained using fluorescence microscopy (Scale bar = 50 μm). **(D-F)** Effects of blue photogenetic stimulation on the firing rate of dopaminergic axonal terminals in dHPC CA1 of Slc6a-iCre$^{+/-}$: Rosa26-ChR2$^{+/-}$ mice. (D) Diagram showing spike distribution with or without photogenetic stimulation. (E) Optogenetic modulation parameters. (F) Statistical analysis on firing rate as presented in D. All data are presented as mean ± standard error of the mean. Student's t-test was performed to compare the differences between two groups (F). **, $P < 0.01$.

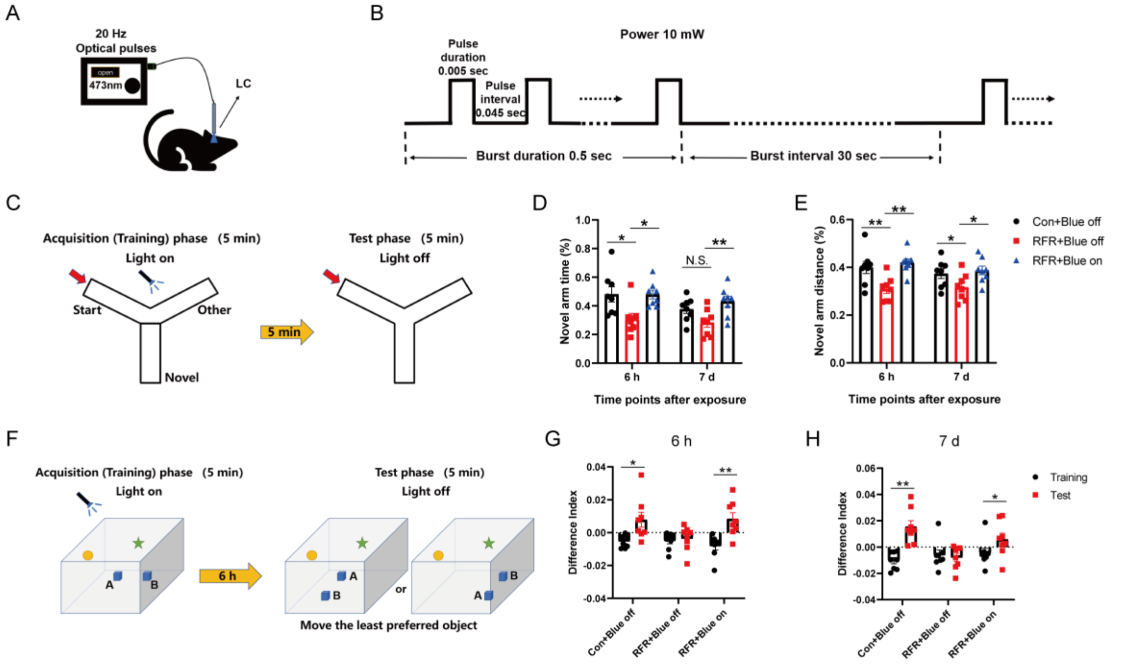

Suppl. Fig. 5 Optical activation of locus coeruleus dopaminergic neurons improved spatial learning and memory impairment in mice caused by radiofrequency exposure. (A) Schematic of the blue optogenetic system. **(B)** Optogenetic modulation parameters. **(C-E)** Performance of mice receiving optogenetic stimulation in Y maze test. (C) Experiment design. (D) Novel arm time (%). (E) Novel arm distance (%). **(F-H)** Performance of mice receiving optogenetic stimulation in spatial object recognition test. (F) Experiment design. (G-H) Difference index each group 6 h and 7 d post exposure. Data are presented as mean ± standard error of the mean. One-way analysis of variance followed by Bonferroni's post hoc test was performed to compare multiple groups (D, E). Student's t-test was performed to compare the differences between two groups per time point (G, H). *, $P < 0.05$; **, $P < 0.01$; N.S., non-significant ($P > 0.05$).

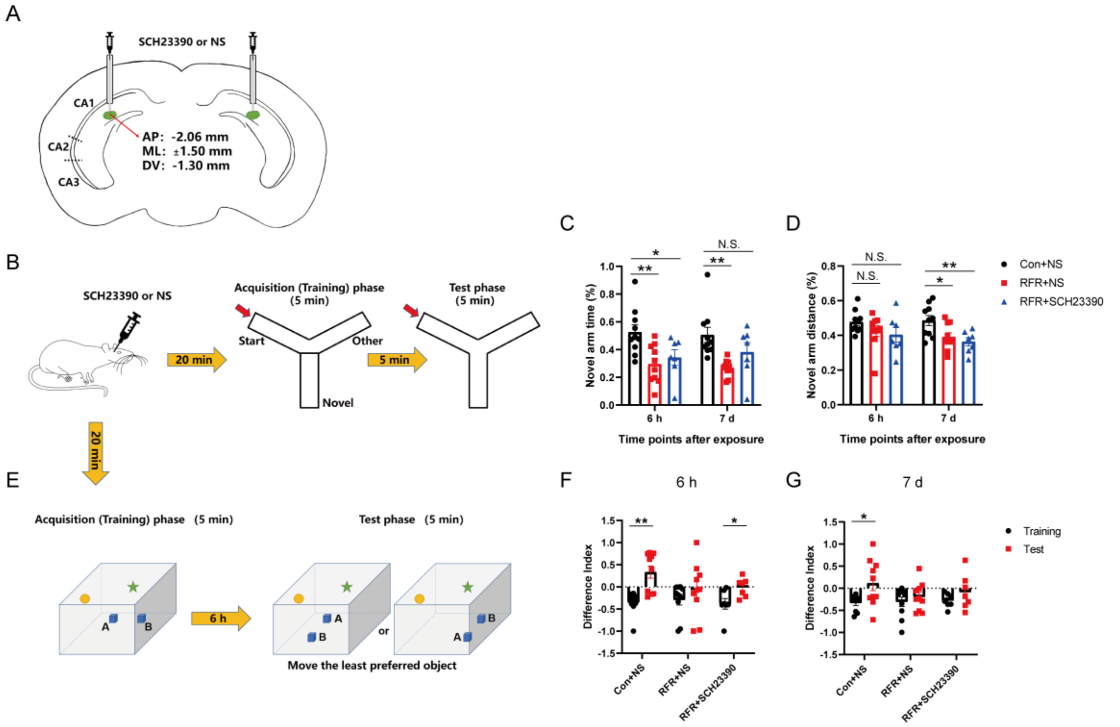

Suppl. Fig. 6 DA receptor antagonists SCH23390 administrated in dorsal hippocampus CA1 impaired the spatial recognition memory in naïve mice. (A) Schematic of drug administration. **(B-D)** Y maze, novel arm discrimination test. (B) Experiment design. (C) Novel arm time (%). (D) Novel arm distance (%). **(E-G)** Spatial object recognition experiment. (E) Experiment design. (F-G) Difference index of each group 6 h and 7 d post exposure. Data are presented as mean ± standard error of the mean. One-way analysis of variance followed by Bonferroni's post hoc test was used to compare multiple groups (C, D). Student's t-test was performed to compare the differences between two groups per time point (F, G). *, $P < 0.05$; **, $P < 0.01$; N.S., non-significant ($P > 0.05$).
